# Shaping public health policy in personalized prevention: the multidimensional PROPHET framework

**DOI:** 10.1093/eurpub/ckag180

**Published:** 2026-10-14

**Authors:** Astrid Moura Vicente, Angelica Valz Gris, Cristina Costa, Maria Luis Cardoso, Alexandra Costa, Fátima Lopes, Pragathy Kannan, Markus Perola, Roberta Pastorino, Angelo Maria Pezzullo, Stefania Boccia

**Affiliations:** Departament of Health Promotion and Non-communicable Disease Prevention, Instituto Nacional de Saúde Doutor Ricardo Jorge, Lisbon, Portugal; BioISI-Biosystems & Integrative Sciences Institute, Faculty of Sciences, University of Lisbon, Lisbon, Portugal; Section of Hygiene, University Department of Life Sciences and Public Health, Università Cattolica del Sacro Cuore, Rome, Italy; Departament of Health Promotion and Non-communicable Disease Prevention, Instituto Nacional de Saúde Doutor Ricardo Jorge, Lisbon, Portugal; Departament of Health Promotion and Non-communicable Disease Prevention, Instituto Nacional de Saúde Doutor Ricardo Jorge, Lisbon, Portugal; BioISI-Biosystems & Integrative Sciences Institute, Faculty of Sciences, University of Lisbon, Lisbon, Portugal; Departament of Health Promotion and Non-communicable Disease Prevention, Instituto Nacional de Saúde Doutor Ricardo Jorge, Lisbon, Portugal; Departament of Health Promotion and Non-communicable Disease Prevention, Instituto Nacional de Saúde Doutor Ricardo Jorge, Lisbon, Portugal; BioISI-Biosystems & Integrative Sciences Institute, Faculty of Sciences, University of Lisbon, Lisbon, Portugal; Faculty of Medicine, Clinical and Molecular Metabolism Research Program (CAMM), University of Helsinki, Helsinki, Finland; Faculty of Medicine, Clinical and Molecular Metabolism Research Program (CAMM), University of Helsinki, Helsinki, Finland; Section of Hygiene, University Department of Life Sciences and Public Health, Università Cattolica del Sacro Cuore, Rome, Italy; Department of Woman and Child Health, Fondazione Policlinico Universitario A. Gemelli IRCCS, Rome, Italy; Section of Hygiene, University Department of Life Sciences and Public Health, Università Cattolica del Sacro Cuore, Rome, Italy; Section of Hygiene, University Department of Life Sciences and Public Health, Università Cattolica del Sacro Cuore, Rome, Italy; Department of Woman and Child Health, Fondazione Policlinico Universitario A. Gemelli IRCCS, Rome, Italy

## Abstract

There is currently no widely accepted framework to guide decision-making for preventive approaches using genetic and genomic technologies. In the context of the PROPHET project, we developed a multidimensional framework integrating Health Technology Assessment and Health Impact Assessment, complemented by a monitoring phase to assess the impact of policies implementing personalized prevention interventions. This integrated approach links technical, clinical, and societal dimensions, supporting prevention strategies that are effective, equitable and sustainable. The PROPHET framework supports policy makers in the equitable and efficient implementation of personalized prevention policies at scale.

## Introduction

Personalized prevention aims to prevent the onset, progression and recurrence of diseases through the adoption of targeted interventions that consider biological information (e.g. genetic and other biomarkers, demographics, and health conditions), environmental and behavioral characteristics, and the socio-economic and cultural context of individuals. This should be timely, effective, and equitable to maintain the best possible balance in lifetime health trajectory [1]. In public health, personalized prevention shifts from uniform, population-wide strategies toward risk-stratified approaches to optimize population health outcomes, while minimizing avoidable harm and inefficiency.

Despite growing use of personalized prevention approaches [2], comprehensive frameworks to assess the impact of policies implementing such interventions in healthcare remain lacking. Most existing frameworks that assess “omic” technologies are grounded in Health Technology Assessment (HTA) [3, 4]. HTA provides a methodology for evaluating health technologies across multiple dimensions, including clinical effectiveness, safety, cost-effectiveness, organizational impact, ethical, legal, and social implications [5], but is not designed to assess policies or capture broader societal, equity, and distributional impacts. Equity and acceptability are often inadequately addressed in the assessment of genetic and genomic tests [6].

Health Impact Assessment (HIA) offers a policy-oriented approach addressing implementation conditions and broader effects on population health, equity, and healthcare systems [7]. Typically conducted prospectively and stakeholder-informed, HIA informs decision-making to maximize the beneficial effects of policies before implementation. A recent report recommends broader use of HIA to support the translation of genome-based knowledge into public health practice, including integration of genomic information within the HIA process [8].

The PROPHET project addresses the lack of a comprehensive framework to assess both technologies used for personalized prevention and the policies to implement these by developing a multidimensional appraisal framework integrating HTA and HIA, complemented by crucial monitoring of policy implementation.

## Methods

The PROPHET project adopted a multi-phase methodological approach to develop and pilot a comprehensive framework for appraising policies implementing personalized prevention interventions.

First, the project defined indicators for assessing the clinical utility of preventive genetic and genomic tests through a systematic review of existing frameworks, building on previous work by Pitini *et al.* [3]. Overlapping domains were synthesized into 10 core dimensions: acceptability, analytical validity, clinical validity, context, economic impact, equity, feasibility, clinical efficacy, legitimacy, and personal value [6]. To operationalize these dimensions, we conducted a scoping review of formal assessments, systematically collecting and synthesizing the indicators used into a catalogue intended to support future applications, while identifying gaps and inconsistencies and highlighting the need for greater standardization.

In the second phase, we explored the HIA applicability to assess the broader impacts of personalized prevention strategies based on genetic and genomic testing, and its suitability in real-world settings. After reviewing key HIA methodological guidance, a multidisciplinary team, including public health, genomics and policy expertise from Finland, Italy, and Portugal, assessed the transferability of the HIA structure. For framework development and piloting, HIA was applied to three case studies reflecting diverse interventions, health system contexts and implementation levels: *DPYD* molecular testing before fluoropyrimidine treatment for colorectal cancer patients (Finland, Italy, and Portugal), *BRCA1/2* cascade screening for hereditary breast and ovarian cancer prevention (Italy), and pre-emptive multigene pharmacogenetic testing for life course personalized treatment (Italy) [9].

Lessons learned from these case studies shaped the key features for effective appraisal of personalized prevention policies. We iteratively refined the framework through discussions within the PROPHET consortium and validated it in stakeholder workshops involving health professionals, regulatory agencies, public health experts, and patient representatives.

## Results

### Insights into the evaluation of the clinical utility of genetic and genomic tests

The scoping review on indicators for assessing the clinical utility of genetic and genomic tests highlighted substantial heterogeneity in assessment approaches and persistent evidence gaps [6]. Four main insights emerged.

First, the level and type of evidence required to demonstrate clinical efficacy depends on disease characteristics, preventive intervention, and test features; in some contexts, observational evidence may suffice, particularly when supported by strong premises such as high variant penetrance and well-established treatment effectiveness. Second, dimensions beyond efficacy (acceptability, legitimacy, equity, personal value, and feasibility) are essential to capture real-world effectiveness but are inconsistently assessed, often relying on limited qualitative or mixed-methods evidence. Third, although HTA remains the dominant evaluation framework, assessments often emphasize clinical efficacy and cost-effectiveness, with limited structured engagement of patients, clinicians, and other stakeholders. Finally, most evaluations stop at analytical or clinical validity, leaving substantial gaps in organizational, economic, and equity-related assessment.

### Insights into the health impact assessment of personalized preventive approaches

Across the three case studies, HIA indicated that personalized prevention strategies can improve population health through more targeted and timely interventions. However, effectiveness depended on context-specific implementation conditions. While favorable clinical evidence was necessary, it was insufficient for successful translation into routine practice. Implementation was influenced by multiple, interrelated barriers, including organizational capacity, care pathways integration, variability in referral and follow-up, professionals’ awareness and education, patient literacy, and persistent inequities in access to testing and counselling.

Six recurring lessons emerged.

1. Appraisal must extend beyond clinical evidence, even when clinical utility is established, to include access, organizational feasibility, and equity.
2. HIA provided a structured, context-sensitive approach for both prospective and formative appraisal, supporting identification of vulnerable groups, pathway bottlenecks, and service gaps.
3. Data limitations, particularly incomplete information on patient pathways, outcomes, and costs, constrained assessment and continuous improvement.
4. Incorporating context-specific perspectives was essential for generating recommendations, given the influence of organizational, cultural, and system-level factors.
5. Routine monitoring of access, timeliness, and follow-up was critical to sustain effectiveness and equity over time.
6. HIA was particularly informative for early-stage approaches, like pre-emptive pharmacogenetic testing, by helping identify implementation prerequisites, including infrastructure, workforce capacity, governance, and measurable indicators.

### PROPHET framework structure

The PROPHET framework proposes a sequential, integrated appraisal process combining HTA, HIA, and monitoring to support decision-making on implementing personalized prevention policies (Fig. 1). It guides appraisal from evidence generation to real-world implementation, recognizing that robust clinical evidence alone is insufficient for effective and equitable uptake.

**Figure 1:**
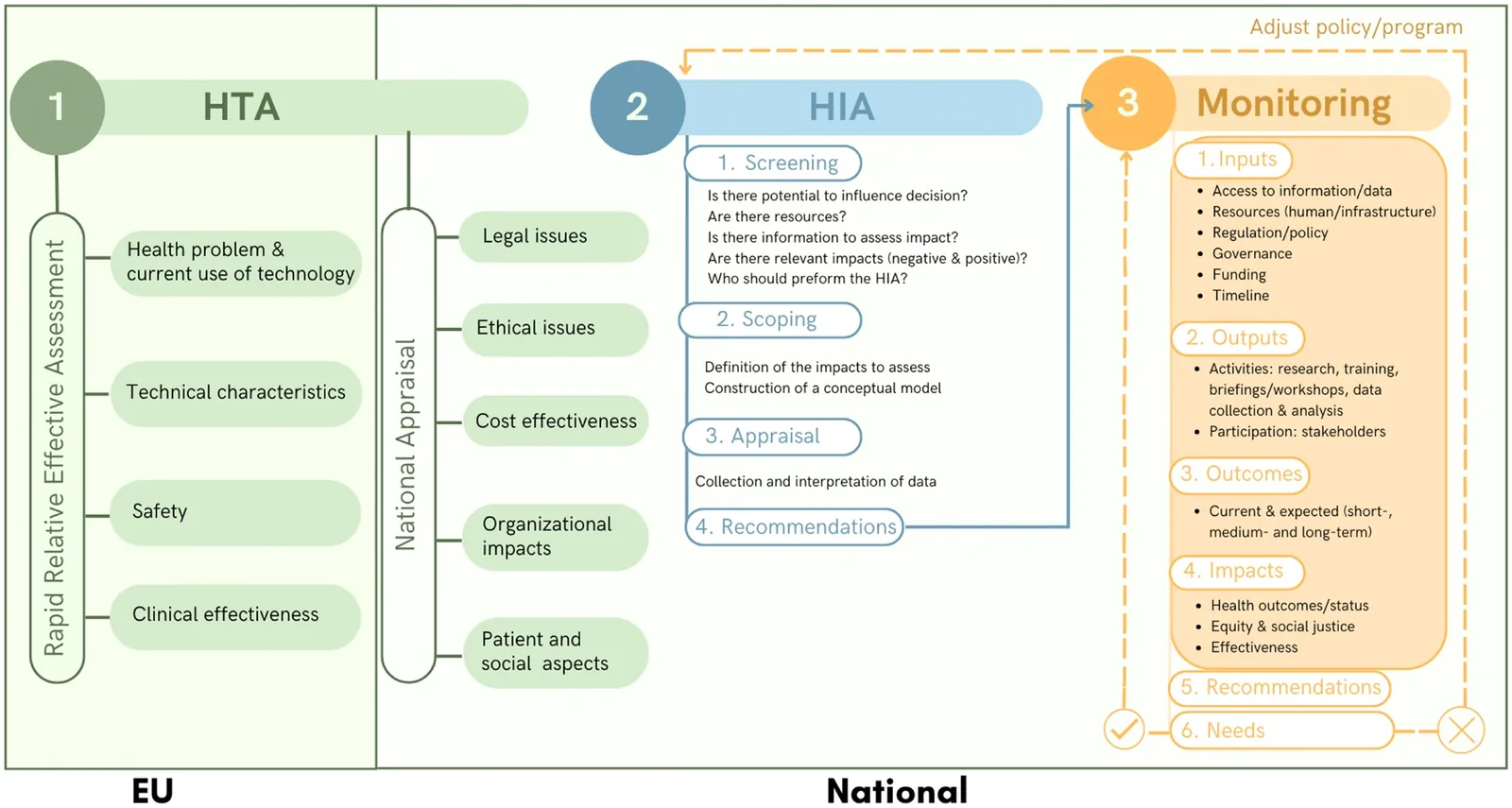
PROPHET framework.

Within the framework, HTA provides an initial assessment of efficacy, safety, and core dimensions of clinical utility, aligned with the European HTA regulation [10] and encompassing joint European-level and context-specific national evaluations. HIA complements HTA by systematically examining the impacts on individual health, services, and health systems of context-specific implementation conditions, including organizational capacity, stakeholder acceptability, and equity-related access. HIA thus identifies barriers and facilitators not captured by HTA alone. Monitoring is embedded as a core component, enabling continuous assessment of access, uptake, outcomes, and equity, and supporting adaptive policy refinement over time.

A potential limitation of the PROPHET Framework is that the sequential integration of HTA, HIA, and monitoring may increase the time and resources required for policy appraisal and may add complexity to already demanding decision-making processes. However, these potential disadvantages should be considered against the added value of a broader and more implementation-oriented assessment. Importantly, the proposed approach does not imply that HTA and HIA should be conducted as fully independent or repetitive assessments. Existing HTA evidence can be reused within the HIA process, while HIA can be proportionate to the characteristics and maturity of the intervention.

## Discussion

To support the implementation of personalized prevention interventions, the PROPHET project developed a multidimensional framework bridging technological assessment and real-world policy implementation. The framework integrates HTA and HIA to support evidence-informed decision-making beyond the technological evaluation alone.

A key feature is HIA, which complements the technology-focused scope of HTA by addressing policy implementation conditions, equity, and broader societal impacts. Through structured stakeholder engagement and embedded monitoring, the framework supports iterative policy refinement and long-term effectiveness. It highlights the importance of multidisciplinary technical capacity, prospective use of HIA with equity focus, reuse of existing HTA evidence, and structured stakeholder engagement. Although the framework may require additional time and coordination, its potential to identify implementation barriers, inequities and unintended consequences at an early stage may reduce the risk of ineffective or inequitable implementation and support more sustainable policy decisions.

In conclusion, the PROPHET Framework provides an integrated approach for appraising personalized prevention policies, extending evaluation beyond clinical utility to feasibility, equity, and population-level impact.

## Data Availability

The data underlying this article will be shared on reasonable request to the corresponding author. Key points The PROPHET Framework integrates Health Technology Assessment and Health Impact Assessment to support the appraisal of personalized prevention policies beyond technology-focused evaluation. The framework extends evaluation beyond clinical utility to inform real-world implementation. The framework provides an implementation-oriented pathway linking evidence generation, decision-making, and continuous learning in European public health systems.

## References

[1] Pastorino R , Pezzullo AM, Osti T et al The PROPHET project paves the way for personalized prevention in the future healthcare. Eur J Cancer Prev 2024;33:387–9. 10.1097/CEJ.0000000000000873 38598497

[2] Farina S , Osti T, Russo L et al The current landscape of personalised preventive approaches for non-communicable diseases: a scoping review. PLoS One 2025;20:e0317379. 10.1371/journal.pone.0317379 PMC11729939 39804869

[3] Pitini E , De Vito C, Marzuillo C et al How is genetic testing evaluated? A systematic review of the literature. Eur J Hum Genet 2018;26:605–15. 10.1038/s41431-018-0095-5 PMC5945588 29422659

[4] Pitini E , Baccolini V, Migliara G et al Time to align: a call for consensus on the assessment of genetic testing. Front Public Health 2021;9:807695. 10.3389/fpubh.2021.807695 PMC8685239 34938716

[5] Kristensen FB , Lampe K, Wild C et al The HTA core model®-10 years of developing an international framework to share multidimensional value assessment. Value Health 2017;20:244–50. 10.1016/j.jval.2016.12.010. 28237203

[6] Pezzullo AM , Valz Gris A, Scarsi N et al A scoping review of the assessment reports of genetic or genomic tests reveals inconsistent consideration of key dimensions of clinical utility. J Clin Epidemiol 2025;181:111729. 10.1016/j.jclinepi.2025.111729 39986491

[7] Harris-Roxas B , Viliani F, Bond A et al Health impact assessment: the state of the art. Impact Assessment and Project Appraisal 2012;30:43–52. 10.1080/14615517.2012.666035

[8] Ádám B , Lovas S, Ádány R. Use of genomic information in health impact assessment is yet to come: a systematic review. Int J Environ Res Public Health 2020;17:9417. 10.3390/ijerph17249417 PMC7765467 33334033

[9] Valz Gris A , Cristiano A, Di Berardino F et al Pre-emptive pharmacogenetic testing in Italy: a review of evidence and multidisciplinary consensus on key priorities for implementation. Front Public Health 2026;14:1763975. 10.3389/fpubh.2026.1763975 PMC13018106 41908762

[10] European Parliament & Council. (2021). Regulation (EU) 2021/2282 of the European Parliament and of the Council of 15 December 2021 on health technology assessment and amending Directive 2011/24/EU. *Official Journal of the European Union*. 2021;L458:1–32. https://eur-lex.europa.eu/legal-content/EN/TXT/? uri=CELEX : 32021R2282 (18 February 2026, date last accessed).

